# Molecular Organization and Patterning of the Medulla Oblongata in Health and Disease

**DOI:** 10.3390/ijms23169260

**Published:** 2022-08-17

**Authors:** Dina Diek, Marten Piet Smidt, Simone Mesman

**Affiliations:** Swammerdam Institute for Life Sciences, University of Amsterdam, P.O. Box 94215, 1090 GE Amsterdam, The Netherlands

**Keywords:** medulla, brain, development, patterning, gene, regulation, Pitt–Hopkins

## Abstract

The medulla oblongata, located in the hindbrain between the pons and the spinal cord, is an important relay center for critical sensory, proprioceptive, and motoric information. It is an evolutionarily highly conserved brain region, both structural and functional, and consists of a multitude of nuclei all involved in different aspects of basic but vital functions. Understanding the functional anatomy and developmental program of this structure can help elucidate potential role(s) of the medulla in neurological disorders. Here, we have described the early molecular patterning of the medulla during murine development, from the fundamental units that structure the very early medullary region into 5 rhombomeres (r7–r11) and 13 different longitudinal progenitor domains, to the neuronal clusters derived from these progenitors that ultimately make-up the different medullary nuclei. By doing so, we developed a schematic overview that can be used to predict the cell-fate of a progenitor group, or pinpoint the progenitor domain of origin of medullary nuclei. This schematic overview can further be used to help in the explanation of medulla-related symptoms of neurodevelopmental disorders, e.g., congenital central hypoventilation syndrome, Wold–Hirschhorn syndrome, Rett syndrome, and Pitt–Hopkins syndrome. Based on the genetic defects seen in these syndromes, we can use our model to predict which medullary nuclei might be affected, which can be used to quickly direct the research into these diseases to the likely affected nuclei.

## 1. Introduction

The medulla oblongata, often shortened to medulla, is a brain structure located at the caudal portion of the brain stem and has an important role in the regulation of various cardiovascular, respiratory, and autonomic functions [1,2]. It is positioned between the pons and the spinal cord, and functions to relay basic but critical regulatory information between the brain and the rest of the nervous system, including sensory, proprioceptive, and motoric information [1]. Being of such vital importance, damage to the medulla can lead to serious problems, including respiratory failure, paralysis, loss of involuntary reflexes (e.g., swallowing, gagging, and sneezing), and loss of sensation (e.g., pain and temperature sensation) [1,2].

The medulla is an evolutionary highly conserved structure that can be identified in all vertebrates, with a similar organization and developmental program [1,3]. It is a complex structure that contains multiple medullary nuclei, densely populated heterogeneous neuronal cell populations, which can be defined based on the expression of evolutionary highly conserved transcription factors (TFs). These medullary nuclei have distinct functions and contain multiple ascending sensory columns, as well as several motor and interneuron populations, each involved in a particular process or functional circuit [4,5,6]. Specific developmental genes have been identified for the development of the medullary nuclei, as well as for some of the various neuronal cell types located outside of these nuclei, which make up the so-called reticular formation [4,6,7]. This suggests that all anatomical (sub-) regions of the medulla exhibit a highly stereotyped developmental logic with a strong underlying genetic organization. Although the neuroanatomy, functionality, and chemical identity of the medulla and medullary nuclei largely overlap between mice and humans, most medullary areas have a more complex architecture and connectivity in humans [8]. However, due to this high structural and functional conservation, the murine medulla provides an excellent study modality to obtain insight in the development and functioning of the human medulla [8]. Understanding the functional anatomy and developmental program of this structure can help elucidate the potential role(s) of the medulla in neurological disorders. Studies described in this review are mainly based on research to the murine medulla. Extrapolation of these findings to humans should, however, still be made with caution.

Much research has been performed to investigate the structure and function of the medulla. However, the details of molecular patterning and how neurons derived from different progenitor domains eventually reach the correct nuclei remain unclear [7,9]. With this review, we aim to provide a complete overview of the existing data on molecular patterning of the medulla during murine development and describe how this patterning ultimately leads to the development of functionally distinct medullary nuclei in the adult medulla.

## 2. Early Developmental Organization

The basis of the medulla is laid down during early mammalian development, when the embryonic central nervous system starts to emerge from the neural plate, which folds into the neural tube [10]. The neural tube expands into three primary vesicles; the forebrain (prosencephalon), midbrain (mesencephalon), and hindbrain (rhombencephalon); the latter being divided along the rostral–caudal axis into 12 molecularly distinct rhombomeres (r0–r11), (r0 defining the isthmus (ist)) [10,11]. The rhombencephalon further divides into two secondary brain vesicles; the metencephalon (prepontine, pontine, and retropontine hindbrain) comprising r0–r6, from which the cerebellum (r0–r2), pons (r3–r4), and retropontine region (r5–r6) arise [12,13], and the myelencephalon (medullary hindbrain) comprising r7–r11, which ultimately make up the medulla [11]. Through further specification of neuronal diversity, and the organizational architecture of neural components, the medulla grows out to a fully functioning adult structure.

**Figure 1:**
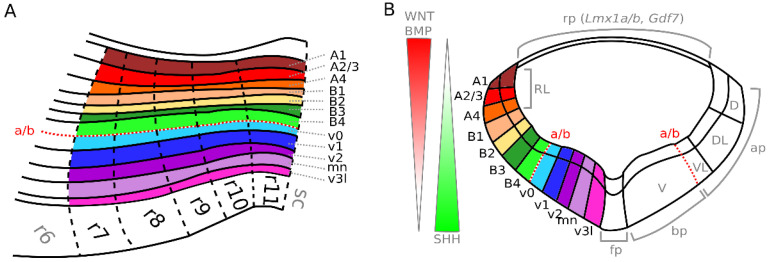
Location of FMUs in the developing medulla oblongata (**A**) Sagittal view of the developing medulla (r7–r11) showing the corresponding FMUs from dorsal to ventral. Different progenitor zones (A1–v3l) are shown with different colors. (**B**) Coronal view of developing the medulla showing the corresponding FMUs. Different progenitor zones (A1–v3l) are shown with different colors. Dorsalizing WNT and BMP signaling from the rp and ventralizing SHH signaling from the fp influence the development of the different FMUs. Expression of *Lmx1a*/*b* and *Gdf7* defines the roof plate. Figures are based on Nieuwenhuys and Puelles (2016) [17] and Di Bonito and Studer (2017) [12]. a/b—sulcus limitans, boundary between alar and basal plate; rp—roof plate; ap—alar plate; bp—basal plate; fp—floor plate; RL—rhombic lip; D—dorsal area; DL—dorsal-lateral area; VL—ventral-lateral area; V—ventral area.

The hindbrain shows a strong genetic organization, formed by the emergence of longitudinal zones and transverse bands (r0–r11), which delimit 3D radially arranged developmental domains, so-called fundamental morphological units (FMUs), each with their own distinct properties and gene expression profiles [1,7]. In the mouse brain, FMUs appear in the hindbrain starting from embryonic day 9–11 (E9–11), under the influence of gradients of morphogens, such as dorsalizing WNT- and BMP-signaling from the roof plate (rp) and ventralizing SHH-signaling from the floor plate (fp), produced by signaling centers located in the neural tube of the surrounding tissue (Figure 1B) [12,14]. Expression of *Lmx1a*/*b* and *Gdf7* are thought to determine rp identity (Figure 1B) [15,16]. These early patterning domains have been observed in embryonic and larval stages of numerous anamniote species, suggesting that all vertebrates share this basic organizational plan and underlining the evolutionary age of this structure [3].

Over time, multiple longitudinal zones have been identified within the vertebrate brain which follow its overall curvature and represent structural as well as functional entities (Figure 1A depicts only the FMUs of the future medulla). At first, the developing hindbrain was thought to consist of two of these homogenous zones. These include the primarily motor *basal plate* (bp), which is ventrally located, and the primarily sensory *alar plate* (ap), located on the dorsal side, the boundary of which (a/b) can be marked within the hindbrain by a ventricular groove termed the *sulcus limitans* [1] (red dashed line in Figure 1A,B). Together, they can be further transversally subdivided into four morphological zones called the *ventral area* (V), *ventrolateral area* (VL), *dorsolateral area* (DL), and *dorsal area* (D) [1]. Their features refer directly to functional and behavioral aspects, corresponding with four functional zones defined over a century ago by Herrick and Johnston, namely: the *somatomotor*, *visceromotor*, *viscerosensory* and *somatosensory columns*, respectively [1] (Figure 1B).

More recently it has been shown that the developing hindbrain can be dorsoventrally divided into at least 16 hypothetical longitudinal domains based on the presence of progenitor cell populations and discrete patterns of gene expression [12,17]. They arise in the developing neural tube as a consequence of dorsoventral patterning by gradients of signaling molecules secreted by the rp (WNT, BMP) and fp (SHH) that, dependent on the concentration and duration of the exposure, control the expression of transcriptional activators and repressors in responding cells (Figure 1B) [12]. Eight alar (dorsal) domains (dA1–dA4 & dB1–dB4) have been described with strong evidence, whereas the eight basal (ventral) domains (v0d, v0d, v0c, v1, v2a, v2b, MN, and v3l) have merely been described based on estimations and are largely homologous to the domains present in the spinal cord (Figure 1B) [4,7].

Within the medulla, 15 of these progenitor domains are present, as it is lacking the dB2 progenitors [4,7]. Each of them has a unique genetic developmental organization that lay the foundation of the generation of diverse neuronal components essential for adult medullary organization and function [18]. These progenitor domains ultimately give rise to multiple genetically distinct neuronal subpopulations with distinct migratory paths, neurotransmitter phenotypes, and axonal projection patterns, thereby contributing to the establishment of neuronal diversity and functional nuclei in the adult medulla [7,11].

As briefly mentioned previously, besides the longitudinal segmentation, the developing hindbrain can be rostral–caudally subdivided into 12 transverse segments along the longitudinal axis, called rhombomeres [13] (r0–r11). These rhombomeres show regional diversity, but share the same primary dorsoventral zones, as a consequence of dorsoventral patterning (Figure 1A) [12,14]. Through the expression of specific combinations of developmental genes that control detailed differential specification, they behave as relatively self-contained proliferative and histogenetic units during the course of development [1]. The 12 rhombomeres of the hindbrain include the isthmus (ist/r0) and 11 rhombomeres (r1–r11), which are distinct developmental regions that correlate with specific hindbrain motor nuclei and nerve roots [11]. Sections r2–r6 can easily be distinguished visually during development as they appear as distinct bulges along the neural tube wall separated by constrictions and ridges [6]. Among other genes, various TFs belonging to the Homeobox (*Hox*) family have been linked to them, which provide a combinatorial code for specifying their unique regional identities [6].

Contrastingly, r7–r11, corresponding to the future medulla oblongata, lack observable outer transverse constrictions, appearing almost continuous with the spinal cord [5,6,19]. The segmentation of this region has proven to be more difficult, and has therefore been strongly debated over time [5,6,19]. Classically, the medullary territory was proposed to either consist of one enlarged r7 segment or a smaller r7 and enlarged r8 segment [5]. The hypothetical boundaries of these rhombomeres, however, lacked cellular and molecular characteristics of typical inter-rhombomeric boundaries [5,6,19]. Furthermore, their size differed significantly from the easily definable rhombomeres 2–6, with the r8 segment being four times larger than the others, suggesting that more segments should be present [5,6].

**Figure 2:**
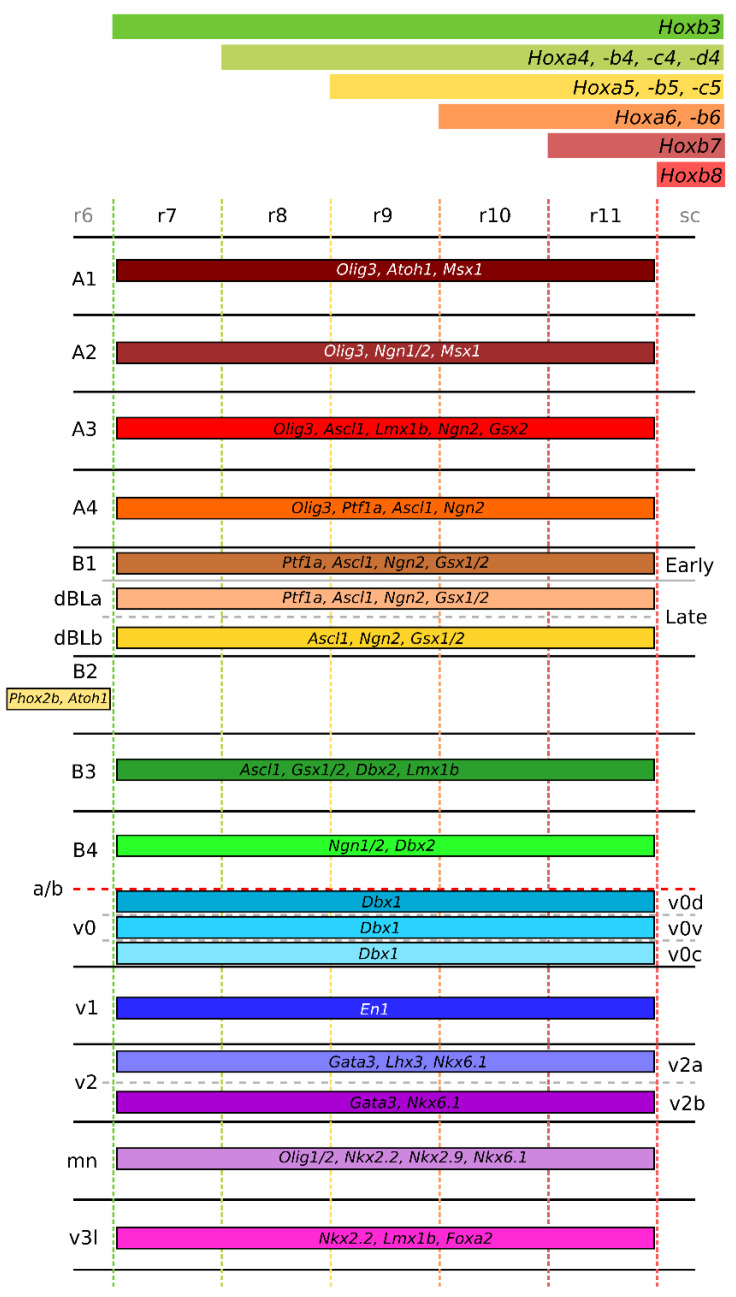
Location and gene expression of progenitor domains in the developing medulla oblongata. Overview of the location and gene expression of progenitors present in the developing medulla. R7–r11 are specified by a unique combination of *Hox*-genes, each combination defining a different rhombomere. Across the rhombomeres, each FMU harbors a unique progenitor population, which can be identified based on a specific combinatorial code of gene expression. Interestingly, progenitors of the dB2 are not present in the developing medulla, although neurons derived from this progenitor domain can be detected in medullary nuclei of the adult medulla. Colors correspond to the colors of the FMUs in Figure 1.

The segmentation of the caudal hindbrain was first examined in chick embryos, which showed that it could actually be subdivided into five different segments, ‘pseudo’- or ‘crypto’- rhombomeres (r7–11), based on their parametric position or on the stepped expression of *Hox* genes, corresponding to morphological transverse limits of a number of medullary nuclei [5,19]. With the use of chick–quail graft experiments, Cambronero and Puelles (2000) showed that neurons born in specific medullary domains become part of distinct medullary nuclei delineated by the boundaries of the pseudorhombomeres. A clear example of this is that, when grafting the r8 segment, the caudal r8 portion of the magnocellular nucleus is made up from these quail-derived neurons, whereas when grafting the r9 segment this nucleus is completely void of these neurons, showing that neurons derived in specific domains are very unlikely to cross the (pseudo-)rhombomeric boundaries [19]. Following avian studies, a similar rostrocaudal segmentation was shown for the murine caudal hindbrain as well, based both on morphological and molecular features [5,6]. The rostrocaudal segmentation of the medulla is highly dependent on the correct expression and combination of *Hox*-genes in the hindbrain [6]. Tomás-Roca and colleagues (2016) showed that the medulla area can be separated in 7 rhombomeres based on the combination of *Hox*-gene expression, which is summarized in Figure 2, similar to avian studies [5]. This resulted in the formation of a more complete segmental map of the hindbrain including all 12 rhombomeric regions (ist/r0–r11), which in turn helped to more precisely locate the position of major structures within the medulla (Figure 2) [5,6,11].

## 3. Molecular Patterning of the Medulla Oblongata and Neuronal Development

As the basic cellular and molecular mechanisms of embryonic hindbrain patterning and development were being unraveled, it slowly became evident that the location of a neuron’s birth is an important deciding factor for its ultimate functional identity [20]. It is now widely accepted that the broad diversity of medullary cell populations results from a combinatorial code of TF expression corresponding to the neuron’s location along the rostral–caudal (R-C) and dorsal–ventral (D-V) axes of the developing hindbrain. Thereby, each progenitor D–V domain produces neurons that share major aspects of neural identity, such as projection pattern and neurotransmitter identity [4,7], and R–C domains define further functional peculiarities or specializations among them [1,12].

This process is regulated by the expression of specifying TFs and signaling molecules during development, that function as instructive signaling molecules that determine the basic developmental characteristics of neurons fundamental to their ultimate functionality, including their anatomical, chemical, and electrophysiological properties [20]. Even though distinct progenitor domains can express similar TFs and signaling factors, each neuronal group is defined by a unique combination of TF and signaling factors, specifying the unique cell-fate and suppressing neighboring fates [7] (Figure 2). The cellular diversity that results from this process is crucial for the correct establishment of functional circuits that ultimately shape the adult medulla oblongata.

The first essential aspect of neuronal specification of the medulla is the establishment of progenitor domains (E9–E11 in mouse) containing relatively large lineage populations with a constrained location extending the entire or part of the length of the hindbrain [1,7]. Once the progenitor domains have been established, their neural derivatives move to their final location through a series of direct and indirect migrations from their specialized *periventricular germinal zones* [20]. Cells destined to become specific motor and sensory deep nuclei of the medulla migrate radially from the *ventricular zone* within their FMU (E9.5–E11.5 in mouse) [20]. During this process, cells from adjacent rhombomeres obtain distinct cellular interaction properties, including differences in cell adhesion and affinity, which segregate them into discrete compartments, so they remain lineage-restricted throughout neurogenesis [1,20]. Within each rhombomere, neurons that originate from a specific dorsoventral division obtain a corresponding function. As only a small set of medullary neurons migrate across rhombomere boundaries, the localization of a significant number of these nuclei is largely consistent with their progenitor domain [20]. 

In contrast, a number of other lineages show overlapping localization with other populations as a result of tangential migration [21]. They are derived from another special proliferative zone called the *rhombic lip* (RL) located at the superior–lateral aspect of the developing brain stem corresponding to part of the alar plate [22,23]. The RL was first defined by His in 1890 based on the morphological features of the hindbrain [24]. Currently, it is thought that the early domain of *Wnt1* expression delineates the RL and that subpopulations of progenitors are further defined by restricted expression of different TFs [24]. From this region, a somewhat later set of long indirect migrations occurs (E11.5–E13.5 in mouse), through which cells leave their respective FMU, causing significant alterations to the anatomy of the medulla [1,13,21]. This results specifically in the formation of a number of so-called *precerebellar nuclei*, to which neurons migrate interrhombomerically [23]. As defined by molecular studies, the RL is divided into three longitudinally arranged microzones, termed A1, A2/3 and A4, based on local expression of *Atoh1*, *Ngn1*/*2* (*Neurog1*/*2*) and *Ptf1a*, respectively [1,25,26,27], which will be further discussed below. These microzones preferentially give rise to precerebellar neuroblasts of a specific migratory stream [1]. Of the more caudal medullary RL derivates, two migratory streams originate; the extensive A1-derived *posterior extramural migratory stream* (ems) and the A4-derived *intramural migratory stream* (ims) [27]. Consequently, even though nuclei that arise from these streams are positioned more ventromedially in respect to their origin, they are considered alar plate-derived and either *Atoh1* or *Ptf1a*-dependent [27,28].

**Figure 3:**
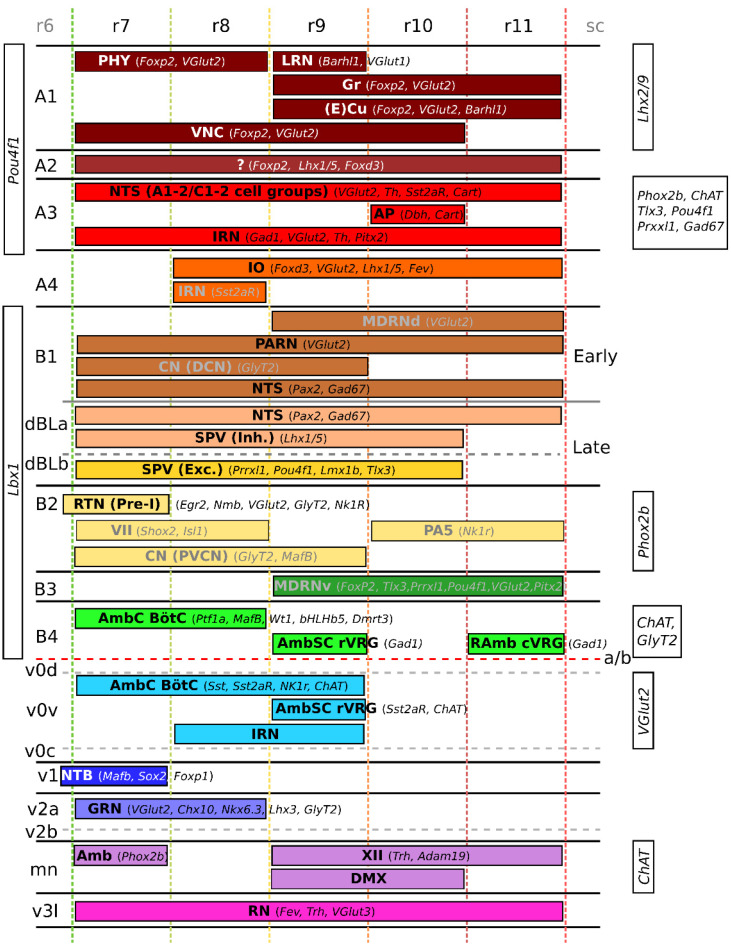
Location and gene expression of medullary nuclei in the adult medulla oblongata. Overview of the location and gene expression of medullary nuclei present in the adult medulla. From each progenitor domain, neurons are formed that belong to specific medullary nuclei in the adult medulla. Colors of the nuclei correspond to the originating progenitor domain. Neurons derived from the dA2 progenitor domain are characterized by the expression of *Foxp2, Lhx1*/*5,* and *Foxd3*, but which nuclei these neurons become part of is currently undefined, as depicted by the ? symbol. Neurons from different nuclei express a unique set of genes, which are highly related to the function of these neurons in the nucleus. Some genes are expressed in neurons of multiple nuclei, shown in the white boxes. Interestingly, some nuclei appear to be build-up from neurons derived from one progenitor domain (e.g., Gr, VNC, SPV, GRN), whereas other nuclei contain neurons derived from multiple progenitor domains (e.g., CN, Amb). The origin of some nuclei is hypothesized and not conclusively shown (depicted in grey).

**Figure 4:**
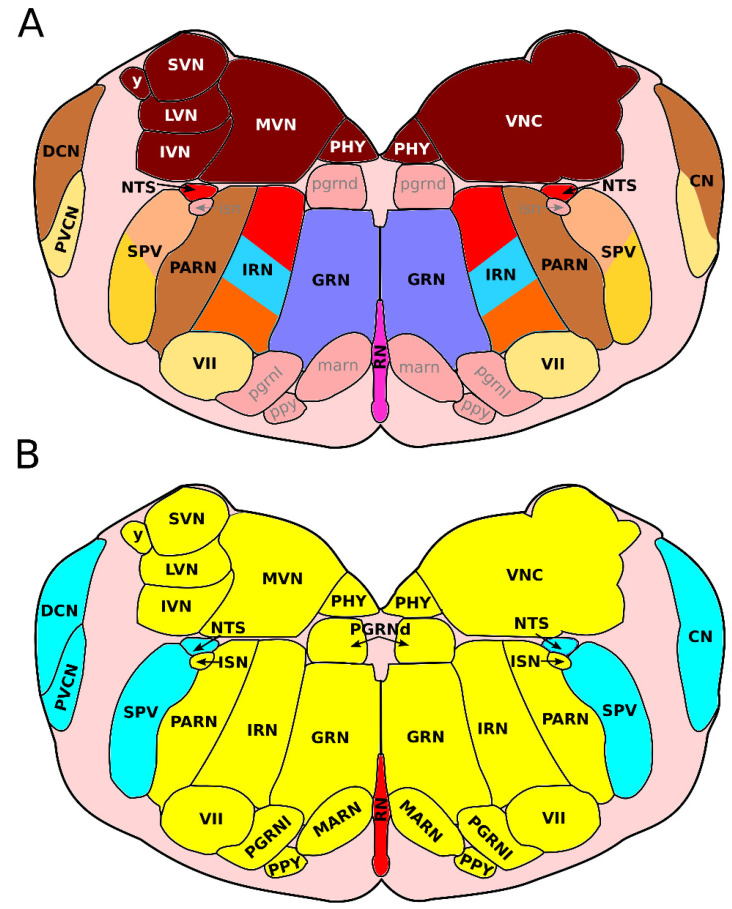
Location of the medullary nuclei in the rostral adult medulla oblongata (r7–r8). (**A**) Location of the medullary nuclei in the rostral adult medulla oblongata. Colors of the nuclei correspond to the colors used as in Figure 3. The origin of the neurons of some nuclei has not been described (e.g., pgrnd, marn, ppy) and are thus shown in grey. (**B**) Overview of the location and overall function of the medullary nuclei in the rostral adult medulla oblongata. Nuclei with a sensory function are depicted in blue, nuclei with a motor function are depicted in yellow, and nuclei with a behavioral function are depicted in red. Overviews are based on anatomical reference atlases from the Allen Brain institute [43].

**Figure 5:**
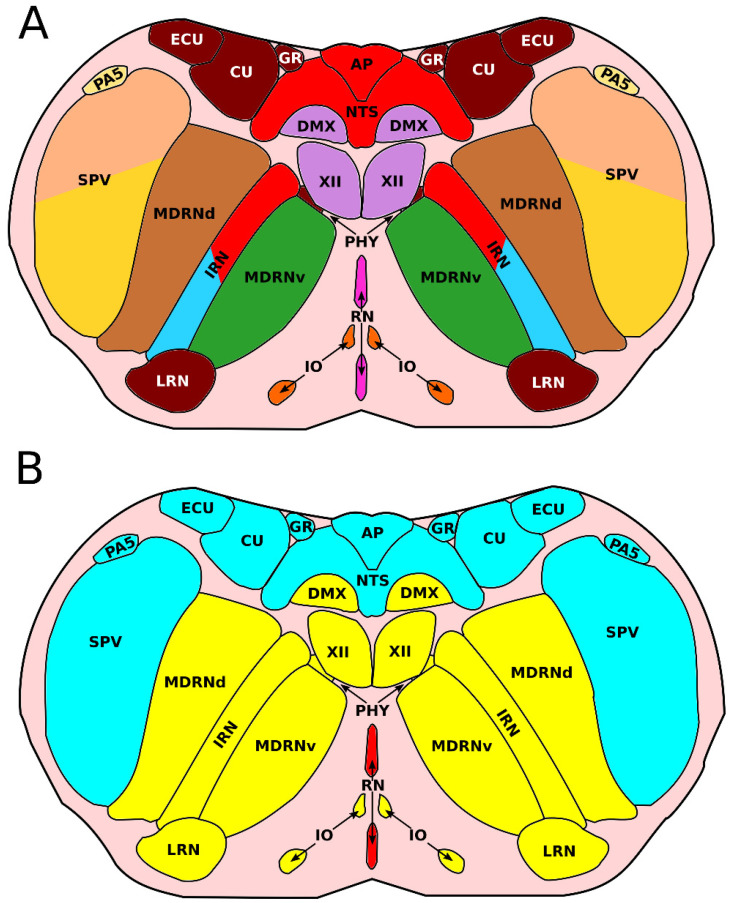
Location of the medullary nuclei in the caudal adult medulla oblongata (r10–r11). (**A**) Location of the medullary nuclei in the caudal adult medulla oblongata. Colors of the nuclei correspond to the colors used as in Figure 3. (**B**) Overview of the location and overall function of the medullary nuclei in the rostral adult medulla oblongata. Nuclei with a sensory function are depicted in blue, nuclei with a motor function are depicted in yellow, and nuclei with a behavioral function are depicted in red. Overviews are based on anatomical reference atlases from the Allen brain institute [43].

The dorsal (or apical) segment of the developing hindbrain involves two sets of progenitor domains: the dorsal-most class A progenitors (present in the RL, Figure 1B), and the more ventrally located class B progenitors [7] (Figure 1). Class A progenitors define four progenitor subdomains (dA1–dA4) through the expression of the basic helix–loop–helix (bHLH) factor *Olig3* [4,29,30] (Figure 2). Of these, dA1 progenitors are further characterized by the expression of *Atoh1* (*Math1*) and *Msx1* [4,25,30,31] (Figure 2). They generate two subpopulations of excitatory glutamatergic proprioceptive relay neurons involved in sensory information processing; one characterized by the expression of *BarHL1* and *VGlut1*, and the other hallmarked by the expression of *Lhx2* and *Lhx9*, *FoxP2,* and *VGlut2* [4,7,25,30,32] (Figure 3). Neurons expressing *BarHl1* and *VGlut1* make up part of the lateral reticular nucleus (LRN) in r9 [7,28,33], involved in motor control in mice [34] (Figure 3, Figure 4 and Figure 5) and the (external) cuneate nucleus ((E)Cu) (r9–r11) [4,7,33,35,36], with the ECu being involved in forelimb proprioception [36] and the Cu involved in tactile feedback modulation of dexterous movement [37] (Figure 3 and Figure 5). Neurons expressing *Lhx2*, *Lhx9*, *Foxp2*, *Pou4f1* and *VGlut2* make up 3 separate nuclei; (1) the gracile nucleus (GR) (r9–r11) [4,7,35], involved in the relay of conscious proprioceptive sensations from the hindlimbs [38] (Figure 3 and Figure 5), (2) the perihypoglossal nuclear complex (PHY) (r7–r8) [4,7], involved in the coordination of tongue movement and autonomic responses to changes in posture [39] (Figure 3, Figure 4 and Figure 5), and (3) the vestibular nuclei (VNC) (r7–r10) [4,5,7,35], involved in body orientation awareness, reflex control of eye movement, and body and head posture [40] (Figure 3 and Figure 4). The VNC is comprised of four different nuclei, namely the lateral vestibular nucleus (LVN), involved in locomotion and postural control [41], the medial vestibular nucleus (MVN), involved in maintenance of gaze and posture [42], the inferior vestibular nucleus (IVN), and the superior vestibular nucleus (SVN) (brain-map.org [43]) (Figure 4). The specific function of the latter two nuclei has not been described, to our knowledge. Interestingly, besides different nuclei in the medulla, the specification of the choroid plexus has also been shown to be dependent on the presence of *Atoh1*-expressing dA1 neurons, as this structure does not develop when *Atoh1* expression is lost [16].

dA2 progenitors co-express *Olig3* with *Ngn1*, *Ngn2*, and *Msx1* [4,7,30,33] (Figure 2). They give rise to excitatory glutamatergic neurons characterized by the co-expression of *Foxp2 Lhx1*, *Lhx5*, and *Pou4fI* [4,7,30,44] (Figure 3). However, it is currently undefined of which nucleus/nuclei these neurons become a part (depicted by a question mark in Figure 3).

dA3 progenitors are characterized by the co-expression of *Olig3, Ascl1*, *Ngn2, Lmx1b, Gsx2* expression [4,7,30,45] (Figure 2). Neurons derived from the dA3 domain are either catecholaminergic or glutamatergic and express the TFs *Tlx3*, *Phox2b, Pou4f1, Prrxl1*, *Gad67* and *ChAT,* the latter two are both involved in neurotransmitter identity, [4,7,9,46,47,48] (Figure 3). These neurons make up the nucleus of the solitary tract (NTS) (both the A1/2 (noradrenergic) and C1/2 (adrenergic) cell groups) (r7–r11), characterized by the expression of *VGlut2*, *Th*, *Cart*, and *SST2aR* [4,6,7,20,45,49] and involved in the integration of respiratory and cardiovascular input, and gastrointestinal functions [50] (Figure 3 and Figure 4), the area postrema (AP) (r10), hallmarked by the expression of *Dbh*, and *Cart* [6,51,52] and involved in the regulation of nausea, feeding behavior, and cardiovascular regulation [53] (Figure 3 and Figure 5), and the intermediate reticular zone (IRN) (r7–r11) [7], specifically the rostroventral lateral reticular nucleus (RVL) [7], (not depicted), hallmarked by the expression of *Gad1, VGlut2, Pitx2*, and *Th* [4,7,54] and involved in the regulation of swallowing [55] and part of the airway control network [56] (Figure 3, Figure 4 and Figure 5).

Lastly, dA4 progenitors, which express *Ptf1a* next to *Ascl1, Olig3* and *Ngn2,* are unique for the hindbrain as they cannot be found in the spinal cord [4,7,29,30,57] (Figure 2). A single neuronal population derives from this domain, constituting glutamatergic neurons that co-express *Foxd3*, *Lhx1*, *Lhx5*, *Fev* and *VGlut2* [4,6,7,30] (Figure 3). This neuronal population makes up the olivary nuclei (IO) (dorsal accessory olivary nucleus (IOda), the medial accessory olivary nucleus (IOma), and the primary olivary nucleus (PO)) (r8–r10) [3,4,7,58], involved in balance control and control of automatic movements [40] (Figure 3 and Figure 5). Furthermore, it has been hypothesized that neurons derived from the *Ptf1a* progenitors from dA4 contribute to the *Sst2aR*-expressing population of the IRN, specifically the RVL (not depicted), in r8 [7] (Figure 3, Figure 4 and Figure 5).

The dorsal class B progenitors involve another four progenitor domains (dB1-dB4) (Figure 1). In contrast to the class A progenitors, they cannot be defined by one single TF, but all generate neurons that express Ladybird homeobox 1 (*Lbx1*), which works antagonistically to *Olig3* [4,44,59] (Figure 2 and Figure 3). Of these progenitor domains, dB1, dB3, and dB4 progenitors are present in all rhombomeres, and dB2 progenitors are present in rhombomere 2 to 6 [4,44,59] (Figure 2). Since in this case rhombomere 7 corresponds to the medulla, as defined by the traditional rhombomeric segmentation of the hindbrain, progenitors of the dB2 domain are the only ones not found in the medulla when not accounting for neuronal migration [20].

The most dorsal progenitors of the dB1 domain are further defined by the co-expression of *Ascl1*, *Ngn2*, *Ptf1a,* and *Gsx1*/*2* [4,7,9,26,59] (Figure 2). Neurons derived from this domain during early neurogenesis (E9.5–E11.5 in mouse), assume an inhibitory fate and migrate to various locations within the ventral medulla, where they are thought to modulate local circuits [4,26,44,59]. Iskusnykh et al. (2016) showed that neurons derived from this early dB1 domain make up part of the NTS and express both *Pax2* and *Gad67* [26]. It is hypothesized that these neurons become part of the dorsal part of the medullary reticular nucleus (MDRNd) (r9–r11) [7], characterized by the expression of *VGlut2* [7] and involved in respiratory pattern formation (Figure 3 and Figure 5). This has predominantly been shown in rats [60], although some evidence points to a similar function in mice [61]. Furthermore, these neurons become part of the parvocellular reticular nucleus (PARN) (r7–r11) [7], similarly hallmarked by the expression of *VGlut2* [7] and involved in the regulation of visceral and motor systems [62] (Figure 3 and Figure 4). It has recently been shown by Schinzel et al. (2021) that a subset of neurons found within the dorsal cochlear nucleus (DCN) of the cochlear nucleus (CN) (r7–r9), arise from a *Ptf1a*-expressing lineage of progenitors and express *Lbx1*, suggesting that these neurons potentially arise from the dB1 progenitor domain [63] (Figure 3 and Figure 4). The DCN is involved in the integration of non-auditory with auditory stimuli [64], and is hallmarked by the expression of *GlyT2* [65] (Figure 3)

During late neurogenesis (E11.5–E13.5 in mouse), the dB1 progenitor domain expands to generate a mix of two late progenitor domains, namely dBLa and dBLb, of which dBLa progenitors are specifically characterized by the expression of *Ptf1a* [4,7,26,59]. Neurons derived from these progenitor domains make up the inhibitory and excitatory interneurons of the spinal trigeminal nucleus (SPV) (r7–r10), respectively [4,26,59] (Figure 3, Figure 4 and Figure 5). dBLa-derived inhibitory neurons co-express *Lhx1* and *Lhx5* and account for many inhibitory neurons involved in sensory information processing [4,59]. *Ptf1a*-negative dBLb-derived neurons become excitatory and co-express *Prrxl1*, *Pou4fl, Tlx3* and *Lmx1b* [4,44,59]. The SPV receives nociceptive signaling from the head and somatosensory signaling [64,66]. Furthermore, dBLa-derived neurons become part of the NTS, next to the neurons of the dB1 domain, and specifically express *Gad67* and *Pax2* [26].

As stated previously, progenitors in the dB2 domain, expressing *Phox2b* and *Atoh1*, do not arise in the rhombomeres of the future medulla, but can be found in r2–r6 [20,59,67] (Figure 2). These neurons migrate and become part of medullary nuclei (Figure 3). Neurons of the ventral cochlear nucleus (PVCN) (r7–r9) [51]) are thought to arise from progenitor zones outside of the medulla (r2–r5) and migrate towards their final location in the medulla [68,69] (Figure 2). These neurons are suggested to arise from *Atoh1*-expressing progenitors, indicating that they may arise from the dB2 progenitor zone [35,68,69,70] (Figure 3). The PVCN specifically expresses *Mafb* alongside *GlyT2* [68,70] and is involved in the processing of different auditory stimuli [71] (Figure 3 and Figure 4). It is known that depletion of both *Lmx1a* and *Lmx1b* (*Lmx1a*/*b* double knock out (DKO)) affects the development of all auditory nuclei in r0–r5, which is assigned to the loss of *Atoh1*-expressing neurons from the dA1 progenitor population (Chizhikov et al. 2021, Elliott et al. 2021). It is not clear whether the part of the cochlear nuclei that reside in r7–r11 are also affected by the loss of *Lmx1a*/*b*. However, these data could indicate that these nuclei are predominantly build-up from neurons arising from the dA1 progenitor population, or that the loss of *Lmx1a*/*b* expression also affects the expression of *Atoh1* in the dB2 domain. Furthermore, it is known that Pre-I neurons from the parafacial respiratory group of the retrotrapezoid nucleus (RTN), which lies on the border between the medulla and the pons, are derived from the dB2 domain [7,28,72,73] (Figure 3). This nucleus is involved in chemosensitivity, the drive to breathe, and expiratory rhythm generation and can be identified by the expression of *Nmb, Phox2b,* and *GlyT2* [65,74,75,76] (Figure 3). These neurons express *Egr2*, which also controls the formation of rhombomeric segments 3 and 5, alongside *VGlut2* and *NK1R*, and are therefore suggested to arise from r3 and/or r5 and subsequently migrate caudally toward the ventral medulla, possibly into the B2 region within r7–r11 [72,73,77]. Interestingly, Rose et al. (2009) have shown that neurons from the facial motor nucleus (VII) (r7–r8) [51], expressing *Shox2, Phox2b, Isl1,* and *Lbx1* [73,78] and involved in motor control of the face [78], and the paratrigeminal nucleus (PA5) (r10–r11) [51], expressing *Phox2b, Lbx1,* and *Nk1r* and involved in apneic reflexes and jaw opening [73], arise from *Atoh1*-expressing progenitors (Figure 3, Figure 4 and Figure 5). As these neurons are known to express *Lbx1,* they should arise from class B progenitors, from which we hypothesize that these nuclei originate from the dB2 progenitors in r1-r6, as this is the only known progenitor pool in the b-domain that also expresses *Atoh1* [7].

dB3 progenitors are defined by co-expression of *Ascl1*, *Gsx1*/*2*, *Dbx2* and *Lmx1b* [4,7,44,59] (Figure 2). Neurons derived from this domain become excitatory and are characterized by the expression of *Pou4fl*, *Prrxl1*, *Tlx3*, *VGlut2*, and *FoxP2* [4,7,44,59] (Figure 3). The exact fate of these neurons is not known, but they are hypothesized to make up the ventral part of the medullary reticular nucleus (MDRNv) (r9–r11) [7], which expresses *Pitx2* [54], and functions as a relay for sensory and/or higher-command-related modification of respiration [60] (Figure 3 and Figure 5). Similar as for the MDRNd the function of this nucleus has predominantly been studied in rats [60], although some evidence points to a similar function in mice [61].

Lastly, the dB4 progenitor domain is defined by co-expression of *Ngn1*/*2* and *Dbx2* [4,44,59] (Figure 2). Progenitors of the dB4 domain form glycinergic and GABAergic inhibitory interneurons, hallmarked by the expression of *Ptf1a*, *MafB*, *Glyt2*, *Wt1*, *bHLHb5*, *Dmrt3*, *Gad1*, and *ChAT* [4,6,44,59] (Figure 3). These neurons can be detected in the Bötzinger complex of the compact ambiguus nucleus (AmbC BötC) (r7–r8) [7,20], hallmarked by the expression of *GlyT2* and involved in regulation of expiration [79,80] (Figure 3), in the rostral ventral respiratory group of the semicompact ambiguus nucleus (AmbSC rVRG) (r9) [81,82], characterized by the expression of *Gad1* and *GlyT2* and involved in the activation of inspiratory motor neurons [80,82] (Figure 3), and in the caudal ventral respiratory group of the retroambiguus nucleus (RAmb cVRG) (r11) [81], also hallmarked by the expression of *Gad1* and *GlyT2* and involved in the activation of expiratory motor neurons [80,82] (Figure 3).

Within the ventral hindbrain, another group of progenitor domains are present, which have been described less extensively than the dorsal domains. These include the v0 class, involving the v0d, v0v, and v0c domains, of which the progenitors are characterized by *Dbx1* expression [7] (Figure 2). They are present in all hindbrain rhombomeres and the spinal cord [20] and give rise to a combination of glutamatergic, GABAergic, and cholinergic neurons as well as glia cells [7]. From these progenitor domains, the fate of the neurons derived from the v0v domain is best known, whereas the fate of neurons derived from the v0d and v0c domain is undefined. Neurons of the v0v domain become part of the pre-Bötzinger complex of the compact ambiguus nucleus (Amb) (r7–9, mainly r8), characterized by the expression of *VGlut2, ChAT, SST, SST2aR,* and *NK1R* [6,7,74,82], and involved in inspiratory rhythmogenesis and chemosensitivity [74,80] (Figure 3). Furthermore, they make up part of the AmbSC rVRG (r9) [7,81,82], together with neurons of the dB4 progenitor domain [81,82], and are characterized by the expression of *VGlut2, ChAT,* and *SST2aR* [6,7] (Figure 3). The last population of neurons derived from this area becomes part of the IRN (r8–9) [7,56], together with neurons of the dA3 and dA4 domains [7], hallmarked by the expression of *VGlut2.*

Progenitors of the v1 domain are characterized by Engrailed 1 (*En1*) expression [7] (Figure 2). Marrs et al. (2013) showed that neurons of the lateral nucleus of the trapezoid body (NTB), hallmarked by expression of *Sox2, FoxP1,* and *Mafb*, medial NTB, hallmarked by the expression of *Sox2* and *FoxP1,* and ventral NTB, hallmarked by the expression of *Sox2*, arise from this progenitor domain [70] (Figure 3). This nucleus is located on the border between the pons and the medulla (r6–r7) [51] and is involved in processing of auditory information via direct contact with the cochlea [83].

The v2 cluster can be divided in a v2a and a v2b domain, of which the progenitors all express *Gata3* and *Nkx6.1*, but can be separated based on the co-expression of *Lhx3* in the v2a domain [84,85,86,87,88] (Figure 2). The fate of the neurons derived from the v2b domain remains unclear, but neurons from the v2a domain form the gigantocellular reticular nucleus (GRN) (r7–r8) [84,85], characterized by the expression of *VGlut2, Lhx3, Nkx6.3, GlyT2,* and *Chx10* [7,65,87] and involved in the regulation of locomotion [89] (Figure 3 and Figure 4).

Progenitors from the MN domain express *Olig1* and *Olig2* alongside *Nkx2.2*, *Nkx6.1*, and *Nkx2.9* [7,86,88,90,91,92] (Figure 2). Although *Olig1* and *Olig2* are mainly linked to the development of oligodendrocytes, this domain is known to first generate somatic and visceral *Phox2b*-expressing motor neurons (E9–E10.5) and later oligodendrocytes (E11.5–E13.5) [91,92]. Neurons derived from this progenitor domain are characterized by the expression of *ChAT* and make up the hypoglossal nucleus (XII) (r9–r11) that co-expresses *Trh* and *Adam19* [6,93], involved in the control of tongue muscles and respiratory motor control [94] (Figure 3 and Figure 5); the dorsal motor nucleus of the vagus (DMX) (r8–r11) [6]; involved in the regulation of glucose homeostasis and gastrointestinal function [95] (Figure 3 and Figure 5); and the nucleus ambiguus (Amb) (r7) [6], co-expressing *Phox2b* [91] and involved in the control of respiratory reflexes, swallowing, and cardiovascular regulation [96] (Figure 3).

Progenitors from the v3l (v3-like) domain express *Nkx2.2* and *Lmx1b* (Figure 2) and ultimately become serotonergic neurons of the nucleus raphe magnus (RMg), nucleus raphe obscures (ROb), and the nucleus raphe pallidus (RPa) of the medullary raphe nuclei (RN) (r7–r11) [7,20,97], involved in regulation of behavior [98] (Figure 3, Figure 4 and Figure 5). The v3l domain arises from the same NK2 homeobox 2 (*Nkx2.2*)-expressing region as the MN to generate serotonergic neurons (E11.5 in mouse), whereby *Fox2a* specifically serves as a determinant of serotonergic identity [99].

Although the origin of most nuclei is relatively established, the developmental origin of the parapyramidal nucleus (PPY) (r7–r8) [51], the magnocellular reticular nucleus (MARN) (r7–r8) [51], the inferior salivatory nucleus (ISN) r7 [51], and the paragigantocellular reticular nuclei (dorsal and lateral) (PGRNd/l) (r8–r9) [51] remains unknown. To our knowledge there are currently no known specific genetic markers for the PPY, which is involved in chemosensory control of breathing [100] (Figure 4), or for the MARN (Figure 4), of which the function is also unclear. For the ISN, also, no markers are currently known; however, it is established that it is involved in the integration of information of oral receptors and the control of von Ebner salivary glands [101] (Figure 4). The PGRN is hallmarked by the expression of *Gad67* and *ChAT67* [47]. The dorsal part (PGRNd) is involved in sleep-wake regulation in rats [102], whereas the lateral part (PGRNl) is involved in sleep-wake regulation and heart-rate regulation in rats [103] (Figure 4). Currently there is no information on the function of the PGRNd and PGRNl in mice.

## 4. The Medulla Oblongata in Health and Disease

Much of what we know about the function of medullary nuclei in humans comes from studying medullary infarctions in humans and induced lesions in experimental animals [8,104]. However, it is known that defects in genes involved in the development of the medulla can result in phenotypic consequences related to specific medullary functions. Insight into the molecular background of these disorders and medullary nuclei that may be affected by these genetic defects can help in the development of specific treatments for these types of diseases. Here, we describe the current status of disorders related to medullary dysfunction and/or with a genetic cause linked to medullary development, and show which nuclei may be affected based on what we know from the cell-fate of different groups of progenitors.

Congenital central hypoventilation syndrome (CCHS) has been linked to a genetic defect in 3 genes involved in early medullary development, namely; *Phox2b* [105,106], *Ascl1* [107] and *Tlx3* [108], although *Tlx3* may only be indirectly involved in the development of CCHS via formation of a DNA-binding complex with *Pbx3* [109]. CCHS is a rare disorder that is hallmarked by generally normal ventilation during wake, but alveolar hypoventilation during sleep [110]. Most patients with CCHS have a mutation in *Phox2b* that leads to a loss-of-function protein [105,106]. *Phox2b* is expressed in progenitors of the B2 domain, in neurons of the A3, and B2 domains, and in neurons of the Amb derived from the MN-domain [4,7,20,47,91], whereas *Ascl1* is expressed in progenitors of the A4, B1, and B3 domains [4,7], and *Tlx3* in neurons of the A3, B1 (unique for SPV), and B3 domains [4,7,47]. Although the development of the medulla has not been thoroughly studied in these patients, neurons derived from these domains make-up a large set of nuclei that are involved in regulation of breathing, such as the NTS and IRN from the A3 domain [4,6,7,20], the MDRNd from the B1 domain [7], the RTN and Pa5 from the B2 domain [28,73], the MDRNv from the B3 domain [7], and the Amb from the MN-domain [91,96]. A thorough study of the function of these nuclei and neurons within these nuclei is necessary to obtain more insight in the underlying neuroanatomical deficits within this syndrome and possible therapeutic targets. In mice it has been shown that loss of *Phox2b*-expressing neurons in the NTS results in an impaired hypercapnic ventilatory response and could point to a function of this medullary nucleus in CCHS [111], but this is yet to be shown in human subjects. Other possible effects on the development of medullary nuclei upon loss of *Phox2b* in relation to CCHS have, to our knowledge, not been described

*Msx1* has been linked to Wolf–Hirschhorn syndrome (WHS) [112], a multi-organ syndrome that is hallmarked by intellectual disability, craniofacial abnormalities (wide nose-bridge and forehead), microcephaly, abnormal tooth development, heart defects, and seizures [113]. *Msx1* is expressed in progenitors of the A1 and A2 domains [4,7], which ultimately form the PHY, LRN, GR, (E) Cu, and VNC [4,7,28,36]. These nuclei are mainly important for balance, gait, and proprioception, and cannot directly be linked to the symptoms characteristic for WHS. However, some patients suffer from heart and hearing defects, two processes strongly regulated by medullary nuclei (e.g., by the AP and CN respectively) [113]. The ultimate fate of neurons derived from the A2 domain is currently still unknown and it is possible that these neurons make-up part of the nuclei involved in the regulation of these processes. Further studies to the fate of the *Msx1*-expressing progenitors are necessary to obtain more insight into a possible role for medullary nuclei in WHS.

Lastly, *Gata3* has been linked to a DiGeorge-like syndrome, called hypoparathyroidism, sensorineural deafness, and renal insufficiency syndrome (HDRS) [114], characterized by, amongst others, low levels of parathyroid hormone, hearing loss, renal disease, facial abnormalities, autism, cognitive disabilities, and congenital heart disease [115]. *Gata3* is expressed in progenitors of the V2 domain, which ultimately form the GRN [84,85,87]. However, much is still unclear about which medullary nuclei are build-up from neurons of the different V-domains, so it remains possible that other medullary nuclei involved in hearing and regulation of cardiovascular processes may (partially) be formed from these neurons. Studies to the function of *Gata3* have shown that loss of one allele of *Gata3* leads to morphological degeneration of the cochlea, but has no clear effect on the brainstem, cerebral cortex, or outer and middle ear, suggesting that hearing loss in HDRS is a result of peripheral abnormalities [116,117,118]. However, complete loss of *Gata3* results in major malformations of the spinal cord and brainstem [119], suggesting that the underlying molecular cascade in *Gata3* heterozygous mice may still be affected in the medulla and future research to HDRS could still focus on a possible role for this structure in the phenotypical symptoms of this syndrome.

Next to syndromes with a genetic cause that are already known to be involved in development of the medulla, there are several neurodevelopmental disorders that show symptoms related to medullary function (e.g., sleep-wake cycle, breathing regulation) [120], but the genetic cause of has not clearly been linked to medullary development.

Of these neurodevelopmental disorders, the role of the medulla has been most extensively studied in Rett syndrome. Rett syndrome is a severe X-linked neurological disorder, which is caused by a defect in the *MeCP2* gene [121]. Patients with Rett syndrome have been reported to experience sleepiness throughout the day and suffer from poor normal sleep and have severe breathing abnormalities suggested to contribute to the high incidence of sudden death [122,123]. As these patients suffer from severe brainstem-related symptoms, there have been several studies published examining the effects of *MeCP2* on brainstem development and functioning. *MeCP2* is strongly expressed during postnatal development in neurons of the brainstem, including the pons and medulla [121]. In a mouse model for Rett syndrome (*MeCP2*–deficient) an increased susceptibility for hypoxia in the brainstem has been found that could arrest breathing [124]. However, stimulating 5-HT1A receptors was able to protect against the breathing arrest as a consequence of hypoxia, making them a potential therapeutic target in these patients [124]. Interestingly, oral administration of desipramine, a norepinephrine uptake inhibitor, to *Mecp2*-deficient mice similarly relieves severe apnea displayed by these animals [125], suggesting that different types of neurons, possibly in different medullary nuclei, contribute to the affected breathing regulation seen in these animals. *MeCP2* could affect the development of medullary nuclei and the associated neurons early in neuronal development. In the murine cortex it has been shown that loss of *MeCP2* affects cell fate refinement and causes a delay in neuronal maturation [126], a feat that has also been described for during adult neurogenesis in the murine hippocampus, which is dependent on phosphorylation of S421 in MECP2 [127] and during neuronal differentiation in zebrafish [128]. This function of *MeCP2* could be regulated by its function as a TF or by its function in regulation of the chromatin state of the DNA, resulting in transcriptional repression of genes important for neuronal differentiation [127,129]. Together, this could point to an affected medullary development during early embryogenesis, resulting in a general negative effect on the development of neurons and medullary nuclei related to respiratory control. However, this has, to our knowledge, not been extensively studied in the animal model for Rett syndrome.

Children with trisomy 21 (Down’s syndrome) have been reported to suffer from insomnia and sleep breathing disorder [120,130], which is thought to be related to a more unstable ventilatory control [131]. It has been found that children with Down’s syndrome also display a smaller pons and medulla than age-matched controls [132]. Furthermore, Down’s syndrome cell adhesion molecule (DSCAM), a gene strongly linked to the development of Down’s syndrome, is expressed in the developing medulla [133], which could point to a possible underdevelopment of the medulla in Down’s syndrome and consequently an affected regulation of breathing and sleeping. Unfortunately, not much is known about the developmental and molecular consequences of trisomy 21 on medullary development.

Lastly, patients with Pitt–Hopkins syndrome (PTHS) suffer from severe breathing irregularities, such as hyperbreathing and apnea, which can occur both dependent and independent of each other [134]. PTHS is caused by haploinsufficiency of the E-box protein *Tcf4* gene, which results in a smaller corpus callosum, affected cortical and hippocampal development, and has recently been shown to affect the development of the parafacial neurons (RTN Pre-I) of the medulla [135,136,137,138]. Although the genetic cause for PTHS has been known since 2007, there is very little known of the role of this factor in development of the medulla and medullary nuclei. Cleary et al. have shown in 2021 that heterozygous *Tcf4* mutants show similar respiratory problems as seen in PTHS patients and that *Tcf4* heterozygous animals have an affected development of *Phox2b*-expressing neurons in the RTN Pre-I, which show an increase in *Nav1.8* channels [136]. Blocking *Nav1.8* in brainstem slices of these animals resulted in an increased baseline activity of chemoreceptors in the RTN pre-I, and also improves baseline breathing in the *Tcf4* heterozygous animals [136]. A study by Ekins et al. showed that when *Nav1.8* function is blocked in *Tcf4* heterozygous animals, social behavior, nesting, fear-conditioning, self-grooming, and anxiety is normalized to WT levels in these animals [139]. Although breathing was not studied here, this study and the research of Cleary et al. (2021) show great therapeutic promise for these types of treatment [136,139]. Interestingly, TCF4 is suggested to form functional heterodimers with the bHLH protein ATOH1 in the rhombic lip [140]. *Atoh1* is expressed in progenitors of the A1 progenitor domain within the RL and the B2 progenitor domain, from which the RTN pre-I is formed [7,28,73]. Although the functional interaction between TCF4 and ATOH1 has not been shown in the medulla or in the B2 progenitor domain, an affected interaction of these TFs could be at the base of the affected development of the RTN Pre-I as described by Cleary et al. [136].

Taken together, although many neurodevelopmental disorders show problems in basic brainstem-related behavior, much is unknown about the development of the medulla in these disorders. Insight in the development of medullary nuclei, from progenitor to neuron, could help in understanding where the problems seen in these disorders arise and whether there are other nuclei that may be affected that have been overlooked until now. We strongly suggest the use of a more patterning-based approach to determine which neurons of which nuclei may be affected in these disorders and work from there to improve current therapeutics and pinpoint possible novel therapeutic targets to relieve these severe and impactful symptoms in patients.

## Data Availability

Not applicable.
